# Prenatal diagnosis of familial exudative vitreoretinopathy and Norrie disease

**DOI:** 10.1002/mgg3.503

**Published:** 2018-11-25

**Authors:** Jingjing Liu, Jing Zhu, Jiyun Yang, Xiang Zhang, Qi Zhang, Peiquan Zhao

**Affiliations:** ^1^ Shanghai Jiao Tong University School of Medicine Affiliated Xinhua Hospital Shanghai China; ^2^ Sichuan Provincial People's Hospital Chengdu China

**Keywords:** familial exudative vitreoretinopathy, genetic counseling, Norrie disease, prenatal diagnosis

## Abstract

**Background:**

Both familial exudative vitreoretinopathy (FEVR) and Norrie disease (ND) are hereditary retinal disorders which can cause severe visual impairment and blindness at a young age. The present study aimed to report the use of antenatal genetic testing and ultrasound in the diagnosis and counseling of FEVR and ND.

**Methods:**

Amniocentesis and ultrasonography were performed in high‐risk mothers, with children having FEVR or ND, to predict severe ocular abnormalities.

**Results:**

Case 1: A homozygous *NDP* mutation (c.376T>C, NM_000266) was detected in the proband and his mother. Molecular prenatal analysis of the fetal DNA revealed no mutations. No ocular abnormalities were detected on ultrasonography. The pregnancy progressed uneventfully to a normal outcome. Case 2: A novel heterozygous *FZD4* mutation (c.1010dupA, NM_012193) was detected in the proband and his mother. The same mutation was detected in the fetus, but ultrasonography showed no ocular abnormalities. A healthy baby boy with stage 1 FEVR was born after an uneventful pregnancy. Case 3: Deletions of exons 2 and 3 in the *NDP* were found in the proband and his mother. The same deletion mutation was detected in the female fetus, but the ultrasound scan was normal. The pregnancy progressed uneventfully to a normal outcome.

**Conclusions:**

To our knowledge, antenatal genetic analyses were used in conjunction with ultrasound for the first time, to diagnose FEVR and ND, and predict the postnatal prognoses in at‐risk babies.

## 1 INTRODUCTION

Familial exudative vitreoretinopathy (FEVR) is a rare inherited disorder in which retinal blood vessels do not properly develop. The disorder can be inherited in autosomal dominant, autosomal recessive, and X‐linked recessive patterns (Chen, Battinelli, Fielder, et al., 1993; Crecchio et al., 1998; Fei et al., 2014, 2015 ; Jiao, Ventruto, Trese, Shastry, & Hejtmancik, 2004; Omoto, Hayashi, Kitahara, Takeuchi, & Ueoka, 2004; Poulter et al., 2012; Xu et al., 2014). The primary characteristics of FEVR include poor vascular differentiation and incomplete peripheral retinal vascularization. Subsequent ischemia and hypoxia can result in neovascularization, and variable secondary complications can occur, including vascular leakage, fibrovascular proliferation, falciform retinal folds, and partial or total retinal detachment (Ranchod, Ho, Drenser, Capone, & Trese, 2011). Clinical manifestations of FEVR are highly variable, even between the two eyes of the same patient and among affected members of the same family. Some patients are only mildly affected and have no visual defects, and some patients have complete blindness at birth or become blind during the first decade of life (Pendergast & Trese, 1998). Secondary changes can develop in advanced cases, including cataract formation, iris atrophy with synechiae formation, band keratopathy, and phthisis bulbi. Unfortunately, functional and esthetic results can be disappointing, even after many intricate surgical procedures (Fei et al., 2016).

The *LRP5, FZD4, TSPAN12, *and *NDP* proteins are involved in the Wnt/Norrin signaling pathway, which plays a crucial role in normal retinal vasculature development (Junge et al., 2009; Xu et al., 2004). Mutations in these genes underlie the molecular mechanisms that result in FEVR. Mutations in *ZNF408* and *KIF11* were also recently reported to be related to FEVR pathogenesis (Collin et al., 2013; Li et al., 2016).

Norrie disease (ND) is a severe X‐linked form of congenital blindness that has many of the same ocular manifestations as FEVR (Walsh, Drenser, Capone, & Trese, 2011). However, approximately 25% of patients with ND also have mental retardation and deafness (Warburg, 1971). Mutations in the *NDP *underlie ND pathogenesis (Chen, Battinelli, Hendriks, et al., 1993), and some patients develop phthisis bulbi despite vigorous surgical intervention at a young age (Walsh, Drenser, Capone, & Trese, 2010).

Having the ability to prenatally diagnose FEVR and ND would be of value because of their potentially blinding and incurable nature. However, highly variable expressivity and non‐penetrance make prenatal diagnoses challenging. Additionally, there is little information in the literature on this topic. Here, we describe our experiences with prenatal FEVR and ND diagnosis and relate prenatal findings to postnatal outcomes.

## 2 METHODS

Institutional review board (IRB) approval was obtained from the IRB at Xin Hua Hospital (Shanghai, China), an affiliate of Shanghai Jiao Tong University School of Medicine and Sichuan Provincial People's Hospital. All study conduct adhered to the Declaration of Helsinki. Written informed consent was obtained from included patients and their relatives after in‐depth discussion of the risks and benefits of genetic testing and prenatal diagnosis.

### 2.1 Patients and clinical examination

All included subjects were native Chinese, were born full‐term, and had a normal birth weight. Each proband and his/her available family members were evaluated by a panel of retinal specialists and underwent comprehensive age‐appropriate ophthalmic examination. This included visual acuity measurement, slit‐lamp examination, wide‐field fundus photography or fluorescein angiography (FFA), and indirect ophthalmoscopy (28D lens with scleral depression, if needed). Based on clinical findings, each patient was diagnosed and classified using the FEVR clinical staging system, as fully described by Pendergast and Trese (1998).

### 2.2 Genetic testing, in silico, and co‐segregation analysis

All DNA samples were extracted from peripheral whole blood samples. Targeted next‐generation sequencing was then performed using a custom genetic pediatric retinal disease panel using previously described methods (Li et al., 2016). Identified mutations were validated by Sanger sequencing through family members. The Human Gene Mutation Database (https://www.biobase-international.com/product/hgmd) was consulted to identify reported pathogenic variants. Pathogenic effects of novel missense mutations were predicted using polymorphism phenotyping (PolyPhen‐2, https://genetics.bwh.harvard.edu/pph2/), sorting intolerant from tolerant (SIFT; https://sift.jcvi.org/www/SIFT_enst_submit.html), and MutationTaster (https://www.mutationtaster.org/) programs. The penetrance and genotype–phenotype correlation of each variant were also evaluated using a family segregation study.

### 2.3 Amniocentesis and prenatal sonography

Amniocentesis was performed during subsequent pregnancies at a prenatal diagnosis center at Sichuan Provincial People's Hospital (Chengdu, China). QIAamp DNA Blood MiDi kit (Qiagen, Hilden, Germany) was used to extract genomic DNA from the amniotic fluid sample using the standard extraction protocol. An additional prenatal test for common chromosomal trisomies is usually offered at the same time to provide added reassurance that the fetus is normal. All women underwent routine obstetric ultrasounds at their appointed hospitals. Transabdominal ultrasonography (Voluson E8 GE Healthcare; Milwaukee, WI) was also offered to the women so that the fetal eyes could be thoroughly examined in utero. Ultrasonography was only performed after an informed discussion with the parents about examination benefits and limitations.

### 2.4 Postnatal fundus screening

All babies underwent a thorough fundus examination shortly after birth using wide‐field fundus photography or FFA (RetCam, Clarity Medical Systems, Pleasanton, CA, USA). Indirect ophthalmoscopy (28D lens) was also performed with scleral depression when needed.

## 3 RESULTS AND CASE DESCRIPTIONS

### 3.1 Case 1

**Figure 1:**
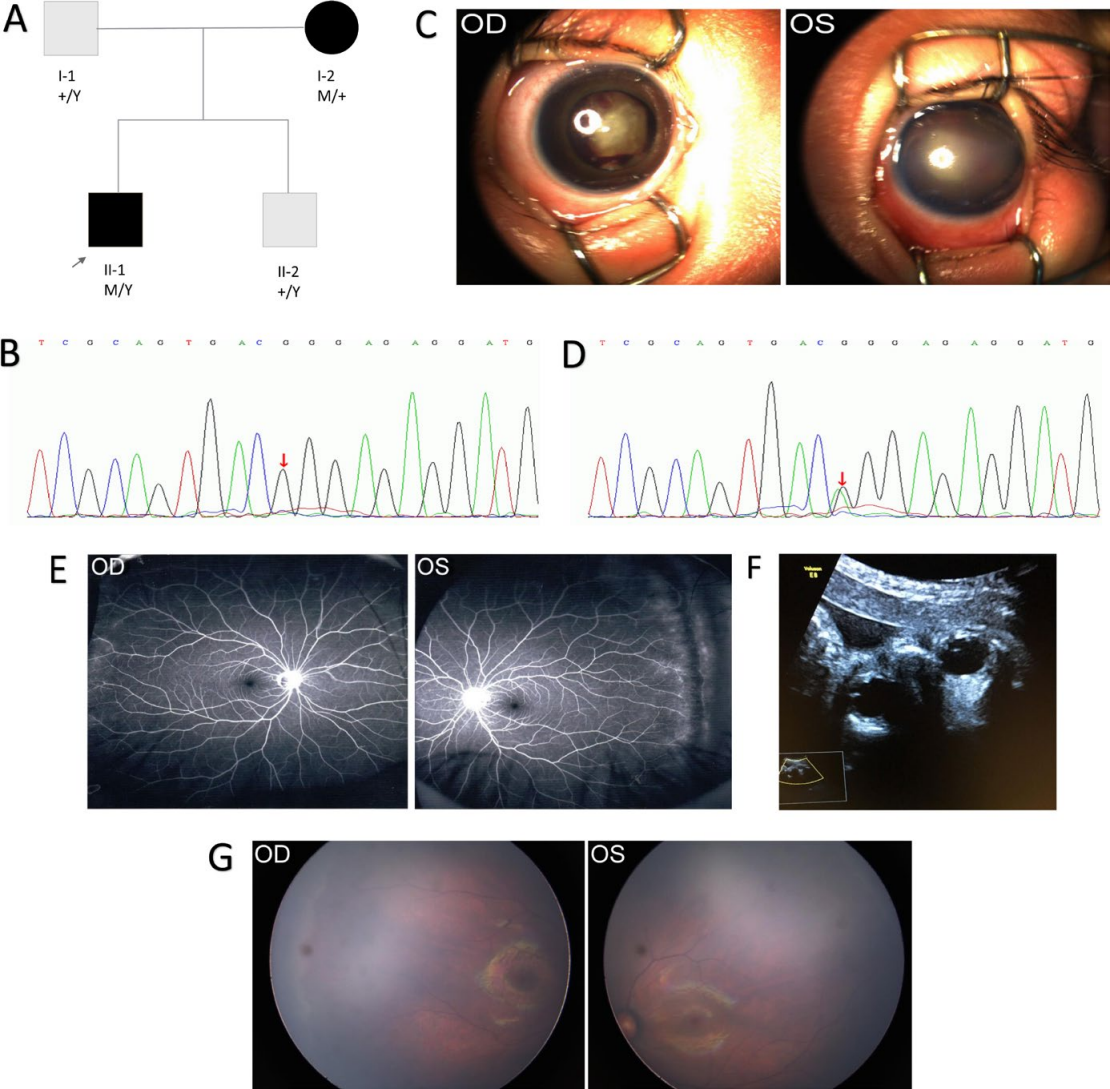
The pedigree, chromatograms, and phenotypes of Family 1. In the pedigree (a): M represents a variant, and +, a normal allele. Y indicates the Y chromosome. In the chromatogram of the proband (b), the variation is marked with red arrows. (c) External photograph of the right eye of the proband shows dense retrolental fibrovascular tissue accompanied by hemorrhage, and the left eye shows a shallow anterior chamber with corneal opacity progressing to buphthalmia. OD and OS represent right and left eyes, respectively. (d) In the chromatogram of the mother, the red arrow indicates the positions of the altered nucleotides. (e) Fundus fluorescein angiography (FFA) image of the proband's mother shows non‐perfusion of peripheral retina and slight leakage. (f) Prenatal ultrasound of the fetus's eyes showed symmetrical ocular globes, transparent and bright lenses, and clear vitreous cavities. (G) Fundus photographs of the newborn baby show normal findings

A 2‐month‐old boy, who was otherwise healthy, was referred to our center for bilateral leukocoria. Retrolental fibrovascular tissue with hemorrhage was found in his right eye. His left eye had a shallow anterior chamber, a corneal opacity, and was progressing toward buphthalmia (Figure 1). Ocular B‐scan showed a closed funnel retinal detachment in both eyes. Genetic testing revealed a homozygous *NDP *mutation (c.376T>C), confirming FEVR. His mother was heterozygous for the mutation. The reported mutation resulted in an amino acid change at codon C126R. This mutation affects cysteine residues responsible for creating the cysteine knot formation, leading to disturbed norrin folding and stability. ND was diagnosed based on the clinical and genetic findings. Ophthalmic examination of the patient's parents was unremarkable, but FFA revealed slight peripheral vascular leakage in the mother.

The mother became pregnant again at 32 years of age and underwent amniocentesis 18 weeks into the pregnancy. No fetal mutations were identified. An ultrasound of the fetus's eyes was also performed at 30 weeks gestational age. Coronal sections showed symmetrical globes, transparent and bright lenses, and clear vitreous cavities. The mother delivered a 3.2‐kg infant at 40 weeks gestational age. Postnatal fundus evaluations were normal in both eyes.

### 3.2 Case 2

**Figure 2:**
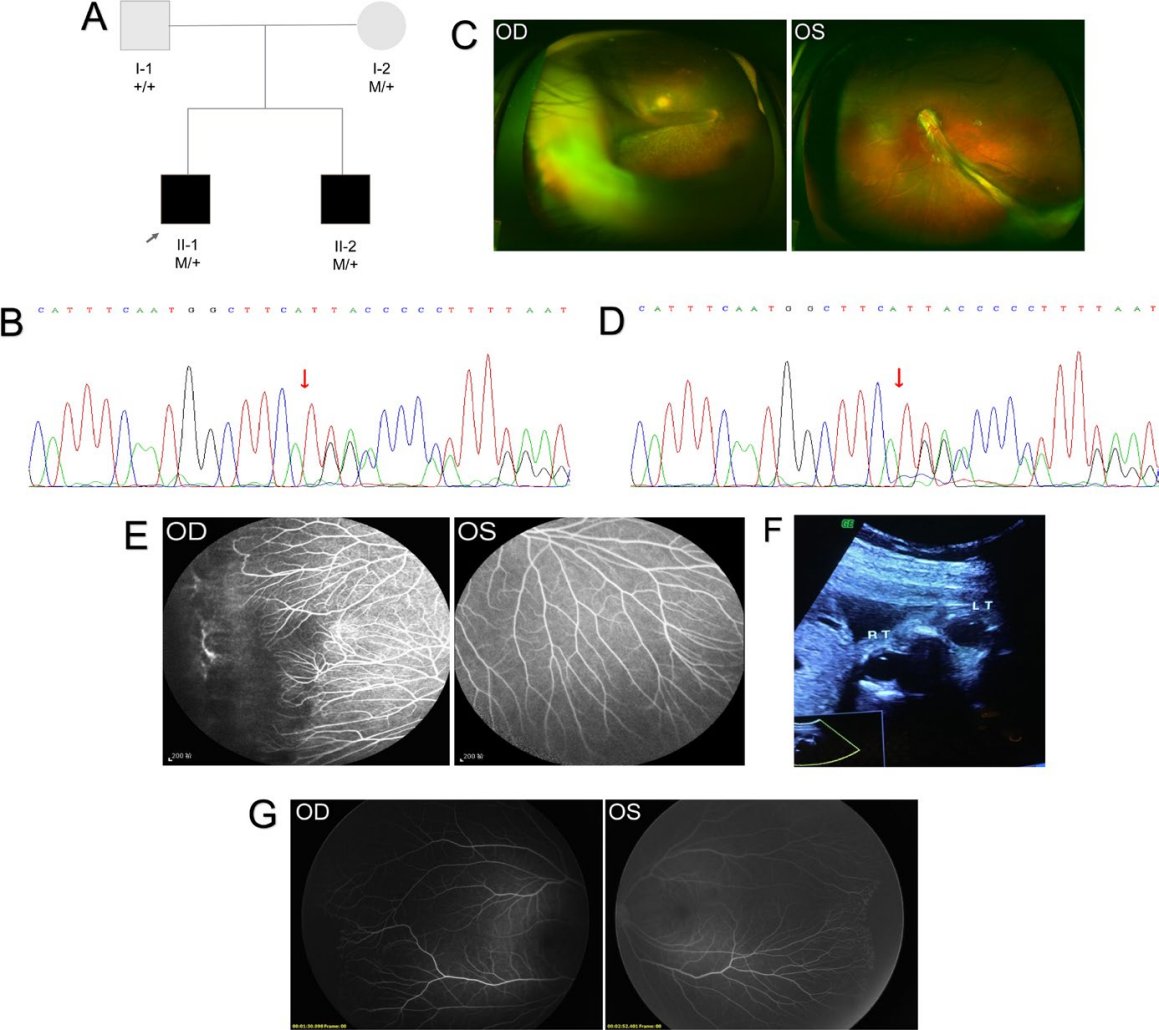
The pedigree, chromatograms, and the phenotypes of Family 2. (a) The pedigree of Family 2. In the sequence chromatograms of the proband (b), the variation is marked with a red circle. (c) Fundus examination showed bilateral retinal folds in the proband of Family 2. (d) In the sequence chromatogram of the proband's mother, the variation is marked with a red circle. (e) Fundus fluorescein angiography (FFA) of the proband's mother shows excessive, straightening vessel branching, vascular leakage, and non‐perfusion area. (f) Prenatal sonography of the fetus's eyes showed symmetrical ocular globes, transparent and bright lenses, and clear vitreous cavities. (g) FFA of the newborn baby shows straightening vessel branching and peripheral avascular zone in both eyes

A 4‐month‐old boy was referred to our center following a routine examination. Fundus examination revealed bilateral retinal folds (Figure 2), along with heavy exudation in the right eye, making him an FEVR suspect. Vision was normal in both parents, and ophthalmologic examination revealed normal anterior segments. Both parents also underwent FFA. The father's results were normal, but the mother had an avascular periphery in both eyes (Figure 2). Genetic testing identified a novel *FZD4* frameshift mutation (c.1010dupA) in both the patient and the mother, confirming the FEVR diagnosis. Disease staging revealed stage 4B and 4A FEVR in the right and left eyes of the proband, respectively, and stage 1 FEVR in both eyes of the mother.

The mother became pregnant for the second time when she was 34 years old and was offered prenatal counseling because the baby had a 50% chance of inheriting the FEVR mutation. An amniocentesis performed at 19 weeks of gestation and revealed that the fetus did carry the *FZD4* mutation (c.1010dupA). The parents decided to continue the pregnancy, and a detailed fetal ultrasound scan was performed at 32 weeks of gestation. No ocular abnormalities were observed. The baby girl was born full‐term at a weight of 3,300 g. Postnatal FFA did not reveal any leakage, but an avascular zone and an excessive, straightened vessel branching pattern was observed in both eyes. Therefore, the baby girl was diagnosed with stage 1 FEVR.

### 3.3 Case 3

**Figure 3:**
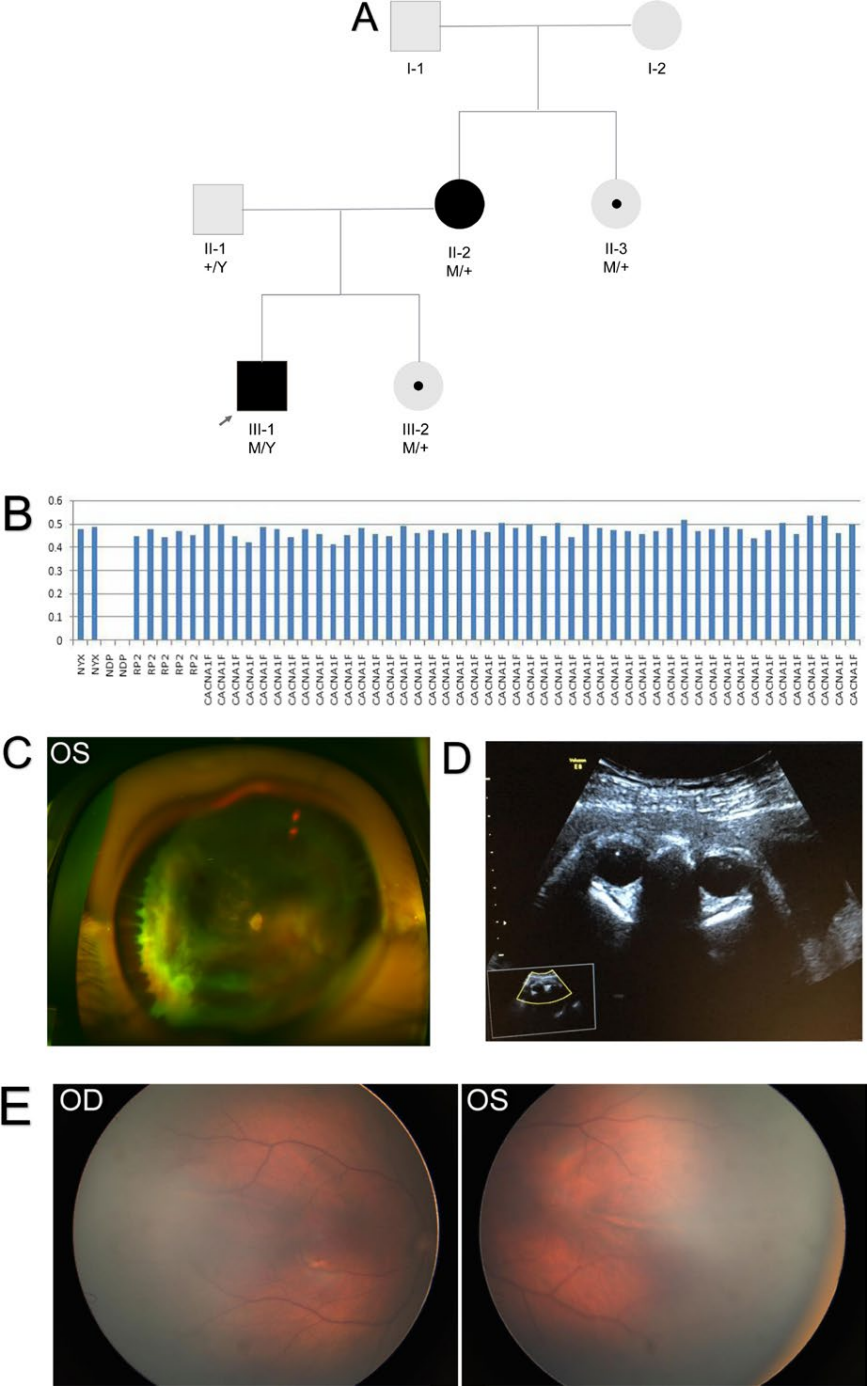
The pedigree and the phenotypes of Family 3 (a). (b) The deletion of two exons was showed (c) Fundus examination shows the partially reattached retina in the left eye of the proband after vitrectomy and lensectomy. (d) Prenatal sonography of the fetus's eyes showed symmetrical ocular globes, transparent and bright lenses, and clear vitreous cavities. (e) Fundus photographs of the newborn baby show normal findings

A 4‐year‐old boy was referred to our clinic for bilateral cataracts and poor vision. His parents reported that he had normal physical and mental development until 2 years of age. Since then, the boy began showing difficulties in learning and communicating with others. Oculo‐digital sign and self‐injury behaviors were also noted. Ocular B‐scan ultrasonography revealed a retinal detachment in both eyes. The patient underwent pars plana vitrectomy and lensectomy in the left eye, and fundus photographs taken after surgery showed a partially attached retina (Figure 3).

The patient's father was healthy, except for physical disabilities related to poliomyelitis. Ophthalmic examination revealed no abnormalities. The mother had phthisis bulbi and keratopathy in the left eye with a visual acuity of no light perception. In the right eye, she had a dragged disk and a visual acuity of 20/125. Genetic testing identified a homozygous deletion of exons 2 and 3 in the *NDP *in the patient. His mother and maternal aunt were both carriers of the deletion. As a result, the proband was diagnosed with ND. Even though the mother was a manifesting ND carrier, the aunt had a normal fundus examination in both eyes.

Prenatal counseling was offered to the couple when the mother became pregnant again because the odds of passing the identified mutation to the child were 50%. Because ND is an X‐linked mutation, a baby boy with the mutation would develop ND. However, a baby girl with same mutation may or may not be a manifesting carrier. The mother underwent amniocentesis at a gestational age of 19 weeks, and genetic analyses revealed that the female fetus had the same *NDP *mutation. The pregnancy was continued, and ultrasound examination at 31 weeks gestational age revealed no abnormalities. A healthy, full‐term baby girl was born with normal fundi (Figure 3).

## 4 DISCUSSION

Both FEVR and ND are inherited retinal disorders in which the molecular analysis of amniocytes is the preferred approach for prenatal diagnosis. However, this method can only diagnose whether the fetus has inherited a genetic mutation from the parent; it is not possible to predict the severity of the condition in an affected pregnancy, because of variability of gene expression. Obstetric ultrasound images were the foundation to understand fetal orbital anatomy, and some studies have reported that standard ultrasound can be used to antenatally detect anophthalmia (Chen et al., 2003), microphthalmia (Leung et al., 2012), retinoblastoma (Lehman, 2003), orbital cyst (Singh et al., 2009), persistent hyperplastic primary vitreous (Esmer et al., 2016), cataract (Ashwal et al., 2016), and retinal detachment (Shen, Zuckerman, Cohen, & Rabinowitz, 2014). Therefore, we advised transabdominal ultrasonography for the pregnant mothers described here to determine the fetal eye development and presence/absence of severe fetal ocular pathologies. Previous studies have reported that a fetal retina that is not attached appears as a conical structure on ultrasound images, with the base at the lens and the apex toward the retina (Farrell, Toi, Leadman, Davidson, & Caco, 1987). Most detached retinas were identified in late pregnancy, between 26 and 37 weeks (Blin et al., 2005; Brasseur‐Daudruy, Vivier, Ickowicz, Eurin, & Verspyck, 2012; Chitayat et al., 1995; Esmer et al., 2016; Farrell et al., 1987; Shen et al., 2014; Wu, Chen, Xie, & Xie, 2017). Therefore, we scheduled ocular ultrasound assessments during this period.

In the first case presented here, the 2‐month‐old male proband was diagnosed with ND. The pathogenic mutation was a previously reported missense mutation (Musada et al., 2016). This mutation affects norrin folding and stability via disruption of key disulfide bonds in the molecule and is associated with a severe phenotype (Wu, Drenser, Trese, Capone, & Dailey, 2007). The boy's mother was the mutation carrier; she exhibited normal visual function and slight leakage in peripheral retina on FFA. Ultrasound findings and fetal genetic analyses were supportive of a normal baby, which was confirmed with a postnatal eye examination. In this case, we confirmed the feasibility of prenatal ultrasound in evaluating the condition of fetal eyes.

The proband in case 2 had poor vision and retinal folds in both eyes. His mother had normal vision, but did have a peripheral avascular zone. A novel pathogenic *FZD4 *mutation (c.1010dupA) was identified in both the proband and his mother. The frameshift mutation resulted in a premature stop codon and a truncated protein. Disease severity was variable in this family. Although the proband's unborn sister also carried the mutation, no abnormal findings were noted on ultrasonography, suggesting that the fetus would have a less advanced form of FEVR. This supposition was confirmed with a postnatal fundus examination, which only revealed stage 1 FEVR in both eyes. Using molecular analysis of amniocytes is an accurate and minimally invasive way to obtain genetic information about a fetus early in pregnancy. However, this method alone is inadequate for prenatally diagnosing FEVR because of the complexity of the condition. Up to 26% of mutation carriers have non‐penetrance (Boonstra et al., 2009), and family members often have varying degrees of disease severity (Pendergast & Trese, 1998; Ranchod et al., 2011; Yuan et al., 2017). Additionally, mutation severity does not seem to be correlated with phenotype severity. Mutations in *FZD4*, *NDP*, *LRP5*, *TSPAN12*, *KIF11,* and *ZNF408* have been shown to result in varying degrees of disease severity within the same family and between eyes of the same individual (Collin et al., 2013; Li et al., 2016; Rao et al., 2017; Toomes et al., 2004; Wu et al., 2007; Xu et al., 2014). Therefore, the introduction of prenatal ultrasound to identify severe ocular conditions in utero is of great significance. In this case, the fetus carried the familial mutation. However, ultrasound images indicated no signs of severe ocular pathologies. This information greatly decreases parental anxiety and stress. Previous reports have shown that in utero ND genetic screening is feasible and accurate (Chow et al., 2010; Sisk, Hufnagel, Bandi, Polzin, & Ahmed, 2014). However, prenatal diagnosis of FEVR with molecular analyses has not yet been reported. Therefore, this case is the first to demonstrate the feasibility and practicality of prenatal FEVR diagnoses. In addition, the prenatal ultrasound examinations provided valuable information about presence/absence of severe fetal ocular pathologies which greatly improve prenatal counseling because the chance of passing the familial mutation to the child is relatively high (50%) in autosomal dominant FEVR. However, a lot of mutation carriers do not show any clinical symptoms due to the reduced penetrance and variation in disease severity. Molecular prenatal analysis alone may largely increase the rate of abortion of such babies. The introduction of prenatal imaging could provide important information about the ocular anatomy of these fetuses. If they were normal on antenatal ultrasound, they would likely be normal or low grade FEVR clinically. On the other hand, if they were abnormal, this could hasten the ophthalmic referral for comprehensive evaluation and treatment. Further, in this case, a novel mutation of the *FZD4*, which has a relatively high penetrance but exhibits varying severity in different members of a single family, was revealed.

Fetal mutation analysis conducted in Case 3 revealed homozygous *NDP* deletion (exons 2 and 3) in the proband. ND is a complex, X‐linked, recessive disorder characterized by retinal dysplasia and congenital or infantile blindness. Sensorineural deafness and progressive mental retardation may also occur. The gross deletion of two coding exons, as identified in case 3, has been previously reported in patients with ND (Schuback, Chen, Craig, Breakefield, & Sims, 1995; Yang, Li, Xiao, Guo, & Zhang, 2012) and resulted in a widely variable disease severity. Schuback et al. (1995 identified a patient with this deletion who only had congenital blindness. This was also true for the patient presented by Yang et al. (2012 but sensorineural deafness and mental development were not assessed because the patient was too young to cooperate. Our patient had low vision accompanied by severe cognitive difficulties, suggesting a role of epigenetic or environmental factors in determining phenotype severity. Our patient did not have hearing loss, but this does not usually manifest until the second decade (Schuback et al., 1995). Therefore, patients with ND should be closely monitored to document dynamic symptom changes. Normally, the female carriers of *NDP* mutation are asymptomatic. However, in case 3, the proband's mother, who is a heterozygous carrier, manifested signs of ND. This may be caused by non‐random X‐chromosome inactivation which leads to uneven number of cells with each chromosome inactivated (Kellner, Fuchs, Bornfeld, Foerster, & Gal, 2009; Shastry, Hiraoka, Trese, & Trese, 1999). Ultrasound evaluation of the female fetus's eyes did not reveal any ocular alterations, suggesting that she was also an asymptomatic mutation carrier. Antenatal ultrasound of the fetus's eyes was normal, and ophthalmic examination of the baby girl at birth confirmed that her fundi were normal.

Prenatal ultrasound can be used to detect serious ocular abnormalities in utero, including retinal detachment and secondary phthisis bulbi (Esmer et al., 2016; Fernandez‐Mayoralas et al., 2014) which could occur in advanced form of FEVR or ND. Therefore, in this case series, prenatal molecular analysis along with ultrasound was used to counsel the pregnant women at high risk. These cases confirm the usefulness of intrauterine ultrasound in the detection of ocular structures. In cases 2 and 3, all ocular ultrasound findings were normal despite the detection of a familial mutation. Both babies were born with functional retinas and were either completely normal or had only minimal disease manifestation, as determined via postnatal fundus examination. Therefore, prenatal ultrasound findings may be indicative of ocular development and a powerful supplement to the prenatal molecular analysis, providing valuable information that is useful to both clinicians and patients. Additionally, fetuses with ocular abnormalities detected on prenatal ultrasound often have accompanying systemic abnormalities, including Walker–Warburg syndrome (Ashwal et al., 2016), PHACE syndrome (Fernandez‐Mayoralas et al., 2014), and Fraser syndrome (Leung et al., 2012). Therefore, molecular analysis is also needed for comprehensive evaluation of the fetus. Signs of mild FEVR including peripheral avascular areas and neovascularization cannot be detected on ultrasonography, but can be successfully managed with laser ablation (Chow et al., 2010) and/or intravitreal vascular endothelial growth factor inhibitors (Sisk et al., 2014).

The results of this case series strongly support the use of molecular analysis and detailed in utero serial ophthalmic examinations via transabdominal ultrasonography in mothers who have children with congenital ophthalmic abnormalities; this provides full information at all stages to assist in informed decisions and guarantee best use of genetic testing as a basis for personalized medical management programs, such as early ophthalmology referrals which help comprehensive fundus evaluation and effective treatment planning.

Our study had several limitations. First, both FEVR and ND are rare inherited retinal diseases, so only three cases were included in our evaluations. Second, prenatal ultrasound image quality is heavily dependent upon fetal position. In addition, there are no established criteria to determine the presence or grade of ocular ultrasound abnormalities because current guidelines for fetal face imaging do not include the eyes (Medicine CoPBOatAIoUi, 2016). Therefore, prospective studies with a relatively large sample size and optimum time and frequency of the evaluation of ocular structures are needed to better understand prenatal ocular ultrasound imaging and how abnormalities may or may not predict ocular structure and function at birth.

In conclusion, our method of using amniocentesis to detect a fetal pathogenic mutation and prenatal ocular ultrasound examination provides valuable diagnostic and prognostic information regarding disease presence and severity and offers safe, reliable help in conceiving a healthy child in severely affected families with inherited exudative vitreoretinopathy such as ND and FEVR. In addition, we reported a novel *FZD4 *mutation resulting in autosomal dominant FEVR with varying disease severity in different members of a family.

## ETHICAL STATEMENT

All study conduct adhered to the Declaration of Helsinki. Written informed consent was obtained from all patients or their legal guardians. Institutional review board approval was obtained from the IRB at Xin Hua Hospital (Shanghai, China), an affiliate of Shanghai Jiao Tong University School of Medicine and Sichuan Provincial People's Hospital.

## CONFLICT OF INTERESTS

None.

## CONSENT FOR PUBLICATION

Informed consent was obtained from all participants to publish the study.
